# Clinicians’ Perspectives on the Use of Robotic Dogs in Therapy for Child Sexual Abuse

**DOI:** 10.1007/s40653-026-00907-1

**Published:** 2026-05-22

**Authors:** Tamsyn B. Reyneke, Tiffani J. Howell, Rebekah L. Chapman, Carol A. Ronken, David Butler

**Affiliations:** 1 The Cairnmillar Institute, Hawthorn East (Wurundjeri Country), Melbourne, VIC 3123 Australia; 2 School of Psychology and Public Health, La Trobe University, (Dja Dja Wurrung and Taungurung Country), Bendigo, VIC 3552 Australia; 3 Bravehearts Foundation, Arundel (Yugambeh Country), Arundel, QLD 4214 Australia

**Keywords:** Animal-assisted therapy, Sexual violence, Therapy dog, Robot dog, Robotic animal

## Abstract

Dog-assisted therapy is associated with improved treatment outcomes for children who have experienced trauma, including child sexual abuse (CSA). However, there are limitations to employing live dogs in therapy (e.g., cost, welfare concerns). Research suggests that robotic dogs may be a viable alternative; however, no published accounts have explored the use of robotic dogs in therapy for CSA survivors. This research aimed to qualitatively explore clinicians’ perspectives on the use of robotic dogs in therapy sessions with CSA survivors, in terms of potential effectiveness, benefits, and limitations. Four clinicians from an organisation that provides treatment to CSA survivors used a robotic dog in their treatment-as-usual sessions with CSA survivors aged between 5—12 years and were then interviewed about their experiences. Thematic qualitative analysis of clinician interviews revealed four main themes: (1) client characteristics that influenced their interest in the robotic dog (e.g., age, shyness); (2) clients’ behavioural interactions with the dog (e.g., cuddling, kicking); (3) robotic dog characteristics (e.g., softness, interactivity, and distractibility); and (4) clinicians’ use of the robotic dog (e.g., for psychoeducation, rapport development). The main reported benefits of the robotic dog were: it was a comfort object for clients, it was useful for psychoeducation, and it helped facilitated the development of therapeutic alliances between clinicians and CSA survivors. Shortcomings included distractibility (e.g., unwanted barking) and lack of technological advancement. Our findings provide preliminary evidence that there are advantages to using robotic dogs in therapy for CSA survivors.

## Introduction

Child sexual abuse (CSA) is a form of traumatic experience whereby a child is subject to sexual behaviour, contact, or activity by an adult or another child who, through age or developmental stage, is in a position of power over the victim (World Health Organisation, 2006). Although prevalence rates likely underestimate actual occurrences (Tarczon & Quadara, 2012), a global estimate of CSA prevalence is around 18% for girls, and 8% for boys (Stoltenborgh et al., 2011). Within Australia, approximately 11% of women and 5% of men have reported experiencing CSA (Australian Bureau of Statistics, 2017), with rates being two to four times higher in Aboriginal and Torres Strait Islander children than non-Indigenous Australian children (Bailey et al., 2017).

CSA typically produces lifelong adverse psychological and psychosocial consequences for survivors (Nagtegaal & Boonmann, 2022). Children who experience sexual abuse may show symptoms of complex trauma, which results from prolonged exposure to interpersonal traumas during childhood (Courtois & Ford, 2013). This can cause impairments in attachment, physiology (e.g., impaired brain development), affect regulation, dissociation, behavioural regulation, cognition, and self-concept (for a review, see Cook et al., 2005). Adults who experienced sexual abuse in childhood are at higher risk for mental health disorders including borderline personality disorder, posttraumatic stress disorder (PTSD), and depressive and anxiety disorders (American Psychiatric Association, 2013; Collin-Vézina et al., 2013; de Aquino Ferreira et al., 2018; De Bellis et al., 2013; Hailes et al., 2019). Furthermore, CSA increases the risk of engaging in deliberate self-harm, suicidal ideation, suicide attempts, and suicide (Devries et al., 2014; Lopez-Castroman et al., 2013; Sigurdardottir & Halldorsdottir, 2018). Behaviourally, children who have experienced CSA can develop internalising symptoms such as reduced interest in play and social withdrawal, as well as externalising symptoms such as aggression towards toys and other people, and temper outbursts (Kendall-Tackett et al., 1993; Lewis et al., 2016).

Given the high prevalence and considerable impacts of CSA, treatments for CSA survivors must be engaging and highly effective. The aim of this research was to explore clinicians’ perspectives about the advantages and disadvantages of using robotic dogs in therapy, and to understand whether robotic dogs are a feasible addition to typical treatments for child survivors of sexual abuse.^1^

### Treatments for CSA Survivors

Extensive research has been conducted on treating CSA survivors (Cummings et al., 2012; Narang et al., 2019). Trauma-informed approaches integrating aspects of family, behavioural, and cognitive therapies can be effective (Cohen & Mannarino, 2015; Dorsey et al., 2011, 2017; Murray et al., 2014). Common components of these treatments include cognitive processing, psychoeducation, play, affect modulation, relaxation, graduated exposure to trauma memories, and enhancing safety (Cohen & Mannarino, 2015; Hanson & Wallis, 2018; Landreth, 2023). Unfortunately, CSA survivors often have less successful treatment outcomes than children who have experienced a non-interpersonal trauma (e.g., car crash; Cohen & Mannarino, 2015; Signal et al., 2017). Contributing factors include lower treatment completion rates, with dropout rates being as high as two to three times that of individuals who experienced non-interpersonal trauma (Imel et al., 2013; Murphy et al., 2014). Given that completion of the full treatment process are associated with better treatment outcomes (Meichenbaum, 2017), overcoming these challenges for CSA survivors is crucial. One such possible approach involves animal-assisted therapy.

### Animal-Assisted Therapy

The employment of animals in therapy is known as animal-assisted therapy (AAT), generally defined as any goal-directed approach where an animal is incorporated *meaningfully* into the treatment process (Kruger & Serpell, 2010). Within AAT, the focus of the treatment remains on the non-AAT evidence-based treatment modality (e.g., play therapy), with animals assisting to support the client’s functioning, and supporting them to work towards achieving their goals. AAT has been used in many settings including community mental health, schools and hospitals (Bert et al., 2016; Binfet & Hartwig, 2019), and has increasingly been demonstrated to be effective for survivors of various types of trauma, including CSA survivors (O’Haire et al., 2015). Animals commonly working in AAT include horses, cats, dogs, and farm animals (Hawkins et al., 2019). However, dogs are most common due to their trainability and accessibility (e.g., being more cost effective and easier to care for than larger animals such as horses), and they have been successfully integrated into many treatment modalities (O’Haire et al., 2015).

### Dog-Assisted Therapy

Dog-assisted therapy (DAT) – where a dog plays an integral role in the treatment process alongside usual treatment (Binfet & Hartwig, 2019) – has been proposed as a potential adjunct to traditional treatment approaches to enhance treatment outcomes for CSA survivors. DAT may be particularly beneficial by facilitating the development and maintenance of trust and rapport between clinician and client, improving client engagement in therapy tasks, and enhancing the efficacy of traditional treatment approaches (Germain et al., 2018; Jones et al., 2019; O’Haire et al., 2015). Such findings suggest DAT may be particularly – although not uniquely—beneficial for CSA survivors, possibly due to the calming effect of having a dog in the therapy room (Lefkowitz et al., 2005), and/or the presence of the dog in helping the client to regulate their thoughts and emotions (Parish-Plass, 2008). Indeed, DAT has been effective in reducing symptoms associated with non-CSA forms of trauma experience, including PTSD symptoms, anxiety, and depression (Germain et al., 2018; Jones et al., 2019; O’Haire et al., 2015), and in improving behaviour-related problems (e.g., externalising behaviours) and global functioning (Stefanini et al., 2015). A prominent limitation in the DAT literature, however, is the lack of reporting on the dogs’ specific involvement in the treatment process and the ways in which individual therapeutic activities incorporating the dogs may contribute to improvements.

Similar promising findings have been observed in DAT studies involving CSA survivors (Dietz et al., 2012; Hamama et al., 2011), although Allen and colleagues (Allen et al., 2022) highlighted some potentially negative impacts of dog involvement. The efficacy of DAT for children and young people, including those who have experienced trauma, has been demonstrated by randomised control trials (Hartwig, 2017; Stefanini et al., 2015, 2016), quasi-experimental research (Dietz et al., 2012; Signal et al., 2017), and qualitative research (Lange et al., 2007; Lubbe & Scholtz, 2013). For example, Dietz et al. (2012) reported that CSA survivors who received DAT combined with treatment as usual displayed significantly greater reductions in symptoms of anxiety, depression, anger, PTSD, and dissociation compared to those who received treatment as usual. Moreover, the incorporation of DAT into trauma-focused cognitive behavioural therapy for CSA survivors has been associated with improved treatment adherence, with as many as 90% of participants completing treatment (Signal et al., 2017); Murphy et al. note that, in traditional treatments, drop-out rates have been estimated to be between 40—60% for CSA survivors (Murphy et al., 2014). In summary, DAT may be an effective way to address some of the barriers to successful treatment for many CSA survivors. To understand why DAT shows such benefits, several theoretical explanations can be considered.

#### Theories of Human–Dog Interactions

Three prominent theories propose why human–dog interactions have positive impacts on wellbeing: (i) social support, (ii) biophilia, and (iii) neurobiology. *Social support theory* proposes that dogs can help to facilitate social interactions and relationships between people (Grandgeorge & Hausberger, 2011; Kruger & Serpell, 2010), provide companionship, and ultimately help individuals to develop empathy and trust towards other beings (Wood, 2011). This is consistent with claims that DAT may facilitate rapport between clients and clinicians (Flynn et al., 2020).

The *biophilia hypothesis* suggests people have a yearning to connect with nature and other (non-human) animals (Wilson, 1984), and that by doing so, they experience strong feelings of safety (Flynn et al., 2020; Julius et al., 2013). This concept may explain why people are often more comfortable speaking to dogs about psychological concerns than speaking to people (Evans-Wilday et al., 2018), which is consistent with the preference of some clients receiving DAT to talk to the dog instead of directly to their clinician (O’Haire et al., 2015).

*Neurobiological theory* proposes that human–dog interactions activate the oxytocin system, which in turn can reduce levels of stress and enhance social connection (Carter & Porges, 2016; Heinrichs et al., 2003). This theory may explain why some individuals, including those with trauma experience, experience reduced levels of psychological arousal in DAT; this assists them to regulate their thoughts and emotions when interacting with a dog in DAT (Geist, 2013; O’Haire et al., 2015).

#### Limitations of Dog Assisted Therapy

Despite promising findings involving DAT for CSA survivors, there are several practical limitations regarding working with dogs that may reduce DAT’s efficacy and feasibility (Wilson & Barker, 2003). Specifically, dogs require a significant time and financial commitment regarding training and care (Jones et al., 2019). According to a survey of U.S. dog owners, the average annual cost of caring for a dog is in excess of US$4,500 (Gollub & McGinley, 2024). Clinicians should also ideally be trained to interact with the dog in an appropriate manner and to incorporate them into their therapeutic practice (Animal Assisted Intervention International, 2019; Therapy Animal Standards USA, 2021), which represents an additional cost and time commitment. Welfare must be continuously monitored to ensure that the dog is not overworked, and that clients’ interactions with them are safe and respectful (Animal Assisted Intervention International, 2019; Therapy Animal Standards USA, 2021). Lastly, dogs may not be feasible for clients who have phobias of and/or allergies to dogs (Animal Assisted Intervention International, 2019; Therapy Animal Standards USA, 2021). As such, researchers are beginning to consider ways to overcome these DAT-related barriers.

### Robotic Animal-Assisted Therapy

Robotic AAT may be a way of gaining the clinical benefits of live animals in therapy, while also avoiding some of the limitations associated with live animals, including dogs (Rabbitt et al., 2015). Robotic animals are more affordable than live dogs, costing usually no more than a few hundred dollars, require little training to use, can work for longer periods of time than live dogs during a day (damage notwithstanding), and require no welfare or allergy considerations.

Research on robotic technology has made considerable advancements, with robotic animals becoming more life-like, responsive, interactive, and complex, leading to them having clinically relevant functions within therapy (Kazdin, 2019; Rabbitt et al., 2015). Caregivers and children have indicated that robotic animals are acceptable to use in therapy for young people (Kitt et al., 2021), and both children and teenagers tend to engage with social robots (Björling et al., 2020; Broadbent et al., 2018). When comparing interactions with a live or robotic dog, Barber et al. (2021) found children to show similar enjoyment when interacting with either type of dog and, interestingly, more positive emotions after engaging with the robotic dog compared to the live dog. They found pre-existing biophilia beliefs influenced participants’ enjoyment of the robotic dog (i.e., the more animacy or emotional characteristics attributed to the robotic dog, the more participants enjoyed their interaction). As such, this suggests that the theoretical underpinnings of human-robotic animal interactions may be like those of human–dog interactions. Conversely, more recent research directly comparing robotic animals and real dogs in therapy for children with Down Syndrome or autism found that real dogs were more effective (Van Der Steen et al., 2025). Additionally, young people are likely to attribute dog-like mental characteristics, morality and sociality to robotic dogs and interact with them as they would a live dog (Melson et al., 2009). In short, some of the benefits attributable to live dogs in therapy may be possible to achieve with their life-like robotic counterparts because they are appraised similarly to live dogs, but more research is needed to explain the mixed findings. Interestingly, Burr and colleagues’ (Burr et al., 2023) study identified that most clinicians interviewed believed that robotic AAT would be a beneficial alternative or preliminary intervention for AAT, yet, notably, only 20% of these clinicians had experience incorporating robotic animals in their practice.

Although still in its infancy, the use of socially assistive robots (e.g., robotic animals, including robotic dogs) has been associated with positive mental health outcomes (Aarskog et al., 2019; Kabacinska et al., 2021; Rabbitt et al., 2015). Specifically, the use of socially assistive robots with elderly nursing home residents is associated with psychological benefits (e.g. Robinson et al., 2013), Moreover, whilst research in clinical contexts involving children has been rare, research on children with autism spectrum disorder (ASD) suggests that socially assistive robots may increase their social and communication skills and emotional regulation abilities (Kim et al., 2013; Kozima et al., 2007). Whether such positive outcomes would apply to CSA survivors – or any other clinical context with children—remains to be explored. Indeed, while this research area is promising, there is a noteworthy limitation in that no prior research has examined the efficacy nor the practical application of incorporating robotic dogs in the treatment of CSA survivors.

#### Limitations of the Current State of Research on Robotic Dogs

Despite the apparent efficacy of socially assistive robots in some treatment contexts, to date most research involves nursing home residents or older adults with dementia as the target population (Aarskog et al., 2019; Moyle et al., 2013). Moreover, when exploring the effects of socially assistive robots with children, much of the research has been conducted through controlled experiments in non-naturalistic settings such as laboratories (Kim et al., 2013; Kitt et al., 2021), or in non-therapeutic settings such as schools (Björling et al., 2020; Broadbent et al., 2018). Thus, there is limited research on the use and feasibility of socially assistive robots being incorporated into therapy sessions for children, and no prior research exploring their use within therapy sessions for CSA survivors. Indeed, as with DAT more generally, there is minimal information in the literature explaining exactly how robotic dogs are, and can be, used in sessions, how they impact treatment delivery, or even clinicians’ perspectives of their utility in therapy. Given the central role that clinicians play in the delivery of interventions and children’s recovery after CSA, it is particularly important to understand clinicians’ perspectives of incorporating and utilising robotic dogs in therapy sessions.

### Current Study

In summary, there are significant psychological and physiological consequences of CSA, necessitating effective psychological treatment for these children. Moreover, CSA survivors may experience difficulties engaging in traditional types of therapy, meaning that alternative or additional options should be explored. While the incorporation of DAT into traditional psychological treatments can benefit CSA survivors, several practical limitations to DAT may impact its efficacy and practical implementation (e.g., costs, allergies). The use of robotic dogs has been proposed as a potential alternative or additional option to traditional DAT for CSA survivors, yet it remains unknown how effective this approach will be for CSA survivors, and what the practicalities of incorporating robotic dogs in therapy sessions with this population will be. As such, the current qualitative study aims to address this gap in the literature by investigating clinicians’ perspectives of the incorporation of robotic dogs in therapy for CSA survivors, including potential effectiveness, benefits, and limitations. A qualitative approach was considered ideal for this purpose given it allows for a rich, flexible, data collection experience involving the exploration and/or identification of unanticipated factors that clinicians may reveal, about the potential use of robotic dogs for CSA survivors (Australian Government Department of Foreign Affairs and Trade 2019).

## Method

Ethical approval to conduct this research was granted by the [to add after blind review]. This study was part of a larger project which involved data collection regarding child outcomes over time after receiving robot-assisted therapy, DAT, or treatment as usual. Only findings from the qualitative interview with clinicians will be reported here.

### Participants

Participants were clinicians employed by [clinic name], an Australian child protection organisation providing treatment and support to children who have experienced sexual abuse and their families. Via convenience sampling, participating clinicians comprised four female clinicians who work across three of [clinic name] counselling offices (locations). No other demographic information about the clinicians is available. All [clinic name] clinicians were invited to participate, with those interested provided with additional information explaining the research and a consent form.

The clinicians utilised the robotic dogs in their sessions alongside treatment as usual with participating child clients who had been referred to [clinic name] for treatment for CSA or problematic sexualised behaviours (*N* = 11, *M* ^age at intake^ = 7.31 years, *SD* = 2.15 years, range = 5.21 to 11.33 years; 81.80% female, 18.20% male). No child participants identified as being an Aboriginal or Torres Strait Islander, or as being from a culturally and/or linguistically diverse background, and all were born in Australia. Inclusion criteria for children were being aged between 4 to 12 years. ([clinic name] typically supports children from 4 years of age. Consultation with clinicians prior to study commencement indicated preference for trialling with this 4—12 year olds because adolescents (defined in Australia as from 12 to 24 years) were expected to be less willing to engage with the robotic dog.. Other inclusion criteria included suspected or reported experience of CSA and being fluent in English (allowing for effective communication with English speaking therapists). Exclusion criteria comprised the child or parent/caregiver having a fear or dislike of robotic dogs, to reduce unnecessary negative reactions within the therapy space. Convenience sampling was used, whereby all clients newly allocated to a participating clinician, and who met the inclusion criteria, were invited to participate in the study. Written informed consent was provided by the child’s parent/caregiver. The clinician, parent/caregiver, and child could choose to withdraw from the study at any time. No compensation was provided for children’s or clinicians’ participation.

### Materials

#### Robotic Dog – Joy for All Companion Pet Pup

The 'Companion Pet Pup' made by *Joy for All* is an interactive, battery-powered robotic dog developed by Ageless Innovation (see: https://joyforall.com/products/companion-pet-pup). This dog comes in two designs, ‘golden’ and ‘freckled’. Two ‘golden’ and two ‘freckled’ dogs were used in the current study. The dogs are described by *Joy for All* as having a soft, life-like coat, speakers to imitate dog sounds, and a ‘heartbeat’. These dogs have built-in sensors used to respond to motion and petting and respond to voices using ‘BARKBACK’ technology. They can be muted to avoid audibly disrupting therapy sessions.

#### Demographic Information Survey for Child Participants

A demographics questionnaire routinely used at intake by [clinic name] identified child participants’ age, gender, and cultural background.

#### Interview Protocol Questionnaire

A semi-structured interview schedule of nine main topics was used when interviewing the clinicians about their experience using the robotic dog (see Table 1 for example questions).

**Table 1:** Example topic questions in the interview schedule

Number	Question Prompt
1	What are your overall perceptions of using robotic dogs in therapy sessions with your child clients?
2	How did you and your clients use or interact with the robotic dog in your sessions?
3	How responsive were your clients to the robotic dogs?
4	How did child clients interact differently with the robotic dogs?
5	Do you think the use of robotic dogs in your sessions had any impact on client engagement?
6	How did the robotic dogs contribute to or hinder the realisation of clinical goals for your clients?
7	What do you see as the benefits of using robotic dogs in therapy?
8	What problems or barriers did you experience in using robotic dogs in therapy?
9	Have you had any experience with live therapy dogs? Do you think robotic dogs are comparable?

### Procedure

#### Therapeutic Procedure

Once informed consent was obtained, clinicians attended a meeting with the members of the [clinic] research team to discuss ways in which the robotic dog may be incorporated into their therapy sessions. Content of the meeting was not shared with the external researchers (i.e., authors to add after blind review) to reduce bias when analysing the interview transcripts. Prior to commencing sessions with the child participants, clinicians were given their own robotic dog to use and educated on how it works.

After providing informed consent, the child participants and their caregivers completed the demographic information questionnaire. Child participants received treatment as usual with the robotic dog present in all their sessions. [treatment centre name] therapeutic treatment for CSA is a holistic approach to therapy that is strengths focussed, collaborative, trauma-informed, and tailored to the individual child. The child’s individual needs determined the length of time child participants spent in treatment and their treatment goals.

Clinicians ensured the robotic dog was in their therapy room prior to beginning all sessions with their participating clients. They introduced the robotic dog to their clients during their first session (at various times depending on the presentation and immediate needs of the child in their first session) and obtained consent from the child prior to turning it on. As per typical treatment protocol, approximately 5 min before the end of the session children were notified that the session would be ending soon, thus allowing the client to finish using the robotic dog (or other toys). In all subsequent sessions, the clients were able to interact with the robotic dog freely, at the discretion of the clinician. The clients’ therapeutic goals and clinicians’ therapeutic approaches guided how the robotic dog was used in the sessions. There was no procedure for systematic collection of usage data in sessions, however, due to limitations in ability for session observation or clinician administrative time allocated for such data collation. As such, data on the extent of use in individual sessions or by individual participants is unavailable.

#### Qualitative Interview Procedure

Author 1 conducted semi-structured online interviews with the clinicians using Zoom. Questions were based on the interview schedule (see Table 1) but, due to the semi-structured nature of the interviews, additional questions were asked and discussed in relation to clinicians’ responses, allowing for them to provide a more nuanced and in-depth description of their experience in the study. This nature of questioning provided clinicians with the opportunity to discuss other topics of interest and provided the interviewer with flexibility to explore these topics in more detail. The length of interviews varied, ranging between 14 to 46 min (average duration = 27.50 min).^2^

Interviews were audio recorded, deidentified, and saved on a password-protected device. Interviews were transcribed verbatim using the software Otter.ai, then were manually checked against the audio recordings and corrected by the lead author, a native English speaker. Interview transcripts were edited for conciseness while ensuring the clinician’s original meaning was maintained (e.g., removing “um” or repeated words). Two other members of the research team (to add after blind review) then reviewed the transcriptions to ensure accuracy and readability. These de-identified transcripts were shared with each clinician for confirmation of accuracy. Three clinicians responded affirming the accuracy. The remaining clinician had resigned from [clinic] so was unable to be contacted.

### Qualitative Data Analysis

The transcribed clinician interview data were analysed thematically using Braun and Clarke’s (2006) six steps of thematic analyses. We adopted an inductive approach to this analysis, given its exploratory nature. This ‘ground-up’ approach allowed codes and themes to emerge from the data, rather than attempting to fit the data to an existing theory or perspective. The analysis software Quirkos 2.5.3 was used when generating codes and identifying themes in the data. Microsoft Word was then used when revising and defining themes produced via Quirkos. Braun and Clarke’s six steps were applied as follows.

Step 1, familiarisation with the data: Author 1 listened to the audio recordings of the interviews and re-read each transcript numerous times to ensure adequate familiarity. Moreover, the time spent editing the transcriptions and re-writing them for optimum readability and clarity further improved this familiarity. Two other members of the research team (authors 2 and 5) also reviewed the resulting transcripts multiple times to ensure their own familiarity with the data.

Step 2, generating initial codes: The interview transcripts were systematically worked through in their entirety to generate codes using a line-by-line analysis (Chenail, 2012). An inductive and open-coding approach was used when analysing and generating codes. That is, codes were identified based on content in the transcripts, rather than prior theories, to reflect the clinicians’ words and meaning more accurately (Clarke & Braun, 2013). An inductive approach was chosen due to the lack of prior research in this area and to reduce bias when coding; that is, reduced potential to force the data into preconceived ideas of the authors within inductive approaches (Braun & Clarke, 2006). The constant comparative method of analysing the data was used, whereby each transcript was systematically compared to the others to identify similarities and differences.

Steps 3, searching for themes; 4, reviewing themes; and 5, defining and naming themes: Themes were constructed by evaluating the codes and identifying patterns amongst them. Main themes were then further developed by linking them to sub-themes. To reduce potential researcher bias, this process was completed collaboratively, whereby members of the research team (Authors 1,2, and 5) met weekly to discuss the content of the interview transcripts and codes, and to revise the main themes and sub-themes until a consensus was reached.

### Reflexivity Statement

During the drafting of this paper, all authors self-identified as White Australians. Regarding the issue of CSA, all team members personally feel that this is a crucial social issue warranting attention, with two members (authors 3 and 4) having a professional history of working for an organization assisting CSA survivors. This study hence originated from our shared interest in trying to explore alternative treatment options for effectively assisting CSA survivors. Our interest in robotic animals stems from authors 2 and 5 being trained in animal research, with the former seeing the potential value of dogs whilst working with war veterans, whilst the latter saw the emotional reactions people can have to robotic dogs when living in Japan (e.g., Japanese people grieving the loss of their robotic dogs).

## Results

### General Overview

Clinicians’ therapeutic approaches and number of participating clients varied (see Table 2), as did the degree to which the clinicians had experience using the robotic dog with clients.

**Table 2:** Clinician number, therapeutic style, and number of participating clients^#^

Clinician #	Main Therapeutic Style	Number of Participating Clients
1	Narrative Therapy	3
2	Child-centred play therapy	7
3	Somatic therapy and cognitive behavioural therapy	1
4	Narrative therapy	N/A*

^#^Child demographic information: *N* = 11, *M* ^age at intake^ = 7.31 years, *SD* = 2.15 years, range = 5.21 to 11.33 years; 81.80% female, 18.20% male). No child participants identified as being an Aboriginal or Torres Strait Islander, or as being from a culturally and/or linguistically diverse background, and all were born in Australia

Note. N/A = Not available

*Number of clients seen by Clinician 4 is unavailable as she had withdrawn from the data collection component of the larger project

Clinician 1 reported that her clients were seemingly not interested in the robotic dog, so had less to report than the other clinicians regarding its use in therapy sessions; consequently, she reported the dog did not integrate well into her approach. Clinician 2 reported the robotic dog integrated “seamlessly” into her approach. Clinician 3 had only recently, at the time of interviewing, been allocated a participating client, so was in the early stages of therapy and thought it too early to tell if the dog integrated well into her approach. At the time of interviewing, Clinician 4 had withdrawn from the data collection part of the larger project. However, she was still willing to participate in the interview component of the research to discuss her experience using the robotic dog with clients in their early stages of therapy. She said that the robotic dog was useful for aspects of her approach.

### Interview Results

The thematic analysis of the clinicians’ interviews revealed four main themes regarding robotic dogs: (1) client characteristics that influenced their interest in the robotic dog; (2) clients’ behavioural interactions with the robotic dog; (3) robotic dog characteristics; and (4) clinicians’ use of the robotic dog. As seen in Table 3, there are two to three sub-themes for each main theme.

**Table 3:** Main Themes, Sub-Themes, and Example Quotes from Clinicians Regarding Robotic Dogs^#^

Main Theme	Sub Theme	Example Quote
(1) Client characteristics that influenced their interest in the robotic dog	(i) Age	“The younger ones, in particular, will gravitate towards the dog and be so excited when it turns on and [when] they hear it bark, and [see] its' tail is wagging” – Clinician 2
	(ii) Shyness	“Clients who were quite private [i.e., were shy], so didn’t really like sharing with me any of their disclosures or what happened to them to get them to come to [clinic]…would gravitate towards the dog a lot more” – Clinician 4
	(iii) Diagnoses or symptoms	“The children with autism didn’t notice and didn’t seem to be bothered by it being there or not being there” – Clinician 4
(2) Clients’ behavioural interactions with the robotic dog	(i) Affectionate interactions	“I usually find that… there’s a lot of use of it being a comfort object.” – Clinician 2
	(ii) Aggressive interactions	“She was quite violent with the robotic dog, like would slap it a lot and pick it up by its ears and pull its' ears and kind of be a bit more aggressive” – Clinician 4
(3) Robotic dog characteristics	(i) Positive characteristics	“I think that’s so far my perception is it’s just another toy, but way more interactive.” – Clinician 3
	(ii) Comparison to live dogs	“It eliminates a lot of the barriers to having a real-life dog in therapy” – Clinician 3
	(iii) Limitations	“I think they found it being verbal and making noise when we were talking, a bit of a distraction, and they asked to turn it off.” – Clinician 1
(4) Clinicians’ use of the robotic dog	(i) Passive use/child-led	“My preference is to have the dog in the room turned off and not introduce it, [but] just have it as a dormant thing that they can either approach or not.” – Clinician 3
	(ii) Psychoeducation/education	“What I’ll say [to the child] is, ‘the on button is right down near his bum and we need to ask for consent for… if we can turn him on’ and look at the dog, and I’ll say, ‘Do I have consent to turn your button on’, and then I put the dog up to my ear and [say] ‘he gives consent’.” – Clinician 2
	(iii) Rapport development	“[The robotic dog] has helped… with some young kids to build a rapport in the session.” – Clinician 2

^#^Child demographic information: *N* = 11, *M* ^age at intake^ = 7.31 years, *SD* = 2.15 years, range = 5.21 to 11.33 years; 81.80% female, 18.20% male). No child participants identified as being an Aboriginal or Torres Strait Islander, or as being from a culturally and/or linguistically diverse background, and all were born in Australia

### Client Characteristics That Influenced Their Interest in the Robotic Dog

All clinicians reported on the client factors they think influenced whether the clients were interested in or interacted with the robotic dog.

#### Age

Clinicians 1 and 2 reported the client’s age was a significant factor that influenced their interest in the dog, with Clinician 2 stating that younger clients were more likely to show interest and excitement. Clinician 2 specified that clients who were eight years of age and under seemed to be the most engaged with the dog. The same two clinicians also explained that the older the child was, the less interest they seemed to show in the robotic dog, or other toys in general. Specifically, Clinician 2 reported that clients who were nine years of age and older did not show much interest. Similar sentiments were expressed by Clinician 1, who reported that older clients did not show interest in the robotic dog, other than having some initial curiosity. Notably, some younger children were also reported to show limited interest in the robotic dog, suggesting that other factors also influence their level of interest.

#### Shyness

Two clinicians suggested that children who tended to be shy (or quiet), had difficulties making and maintaining friendships, or who did not easily disclose what had happened to them regarding CSA, were particularly drawn to the dog.

#### Diagnoses or Symptoms

Two clinicians reported that children who had ASD showed less interest in the robotic dog. For example, Clinician 4 reported that the children with ASD would say “that’s not a dog” and would then ignore the robotic dog. It was suggested that, when children with ASD did interact with the dog, their interactions were more aggressive and sensory seeking in nature than neurotypical children’s interactions.

Clinician 1 reported that clients who had decreased attention spans because of trauma seemed to be less engaged with the dog, suggesting that the dog was “not enough” to hold the client’s attention.

### Clients’ Behavioural Interactions with the Robotic Dog

Most clinicians reported on client's interactions with the robotic dog based on those who used it, not those who ignored the robot. Broadly, such interactions can be defined by the themes of affection and aggression.

#### Affectionate Interactions

Three clinicians (excepting Clinician 1) said the dog was often used as a comfort object by clients. They said the dog seemed to help clients to become more comfortable in the space by providing them with the “continuity of having the same thing to come back to” (Clinician 3) and something to own and be connected to in sessions. Three clinicians observed clients being physically affectionate with the dog including patting, kissing, carrying, or cuddling it. Clients were also reported to answer the dog when it barked, as well as talk to and giggle at it. Two clinicians reported that some clients used the robotic dog to direct attention away from themselves in sessions, for example when they were asked about their abuse history or when they were bored in sessions. Clinician 2 reported observing “themes around nurturing [and] caretaking”, stating that clients would pretend to feed, perform surgery on, and put nappies on the robotic dog. It was also reported that clients would listen to the robotic dog’s heartbeat using a medical kit and would always put it back on its mat where it belonged after playing with it.

#### Aggressive Interactions

Some clients were reported to have interacted aggressively or violently with the robotic dog by kicking, throwing, or slapping it. It was reported that clients with ASD diagnoses and neurotypical children interacted with the robotic dog in this way. It was noted that such aggression need not be considered as a negative therapeutic experience.

### Robotic Dog Characteristics

All clinicians reported that characteristics of the robotic dog influenced how they were able to use the robotic dog in sessions. These reports can be categorised into positive characteristics, comparisons between the robotic dog and live therapy dogs, and limitations.

#### Positive Characteristics

Two clinicians reported liking how soft the robotic dog's fur was and how cute it was. Clinician 2 reported not having any issues with using the robotic dog at all. While all clinicians reported that the robotic dog was another toy, it was generally reported to have been more interactive than other toys in their rooms. For example, Clinician 4 reported that her clients paid more attention to the robotic dog than other toy dogs in her room (e.g., non-robotic soft toy dogs). Similarly, Clinician 2 reported that when clients played with her, they would choose the dog for themselves to play with and give her another toy, suggesting that the clients preferred the dog over other toys. It was also reported that the dog was able to be used in a more educational, intentional, and therapeutic way due to its robotic features, compared to traditional toys.

#### Comparison to Live Dogs

While none of the clinicians reported having extensive experience working with live dogs in their therapy sessions, Clinician 3 said that the robotic dog “eliminates a lot of the barriers to having a real-life dog in therapy” (e.g., expense of training therapy dogs, some client's fear of live dogs, and the distractibility of live dogs). Another reported benefit of the robotic dog was that clients are “able to treat the [robotic] dog how they want” (Clinician 4) in the context of being physical with the dog (e.g., to express anger or re-enact situations that the child has experienced, such as physical discipline). It was reported that clients could have learning experiences from these interactions, which they would not be able to do with live dogs for ethical and welfare reasons. However, two clinicians who had previous experience with live therapy dogs reported that clients seemed less engaged with the robotic dog than the live dog. All clinicians agreed that, in its' current state, the robotic dog used was not an adequate substitute for live dogs in their entirety.

#### Limitations

Three clinicians said the robotic dog was occasionally distracting in their sessions due to it barking at inappropriate times which was reported to annoy clients. All clinicians reported that the robotic dog was lacking in technological advancements; specifically, it was not responsive enough. For example, Clinician 2 stated the lack of “genuine” reactions of the dog reduced how it could be used: “…its tail wags whether you kick it across the room or you cuddle it”. Clinician 1 mentioned that the robotic dog was not cuddly due to its weight and the hard, mechanical body underneath the fur.

### Clinicians’ Use of the Robotic Dog

When asked how the robotic dog was used in sessions, the clinicians reported that interactions were mainly child-led (and hence passively used by the clinicians). They reported using the dog as a tool for psychoeducation and rapport development.

#### Passive Use/Child-Led

Clinicians 2 and 3 reported they preferred the client to initiate interactions with the robotic dog, and they would then use the robotic dog as the client showed interest in it. This passive approach of using the robotic dog was also reflected in how and when they introduced the robotic dog to the client for the first time, with Clinician 2 reporting she only introduced the robotic dog to the client if they “showed any interest in it”.

#### Psychoeducation/Education

Three clinicians reported they used the robotic dog as a psychoeducation tool in their sessions. This included using it as a tool to teach their clients about consent, including asking consent from the dog to turn it on (as the on button was near the dog’s bottom), discussing private parts, what interactions need to be consented to (e.g., kissing the robotic dog), protective behaviours, boundaries, and making mistakes. Additionally, Clinicians 2 and 4 said they used the dog to reflect feeling or emotion to clients, for example, reflecting how the robotic dog might be feeling based on how the client was treating it (e.g., when caring for or hitting it). In this way, clinicians reported that the robotic dog assisted clients with their therapeutic goals when used for psychoeducation.

#### Rapport Development

Two clinicians reported that the robotic dog was useful to assist in developing rapport with their client, with Clinician 3 describing the robotic dog as a useful “ice breaker” and “something to mutually talk about that is separate to… [the client] and me”.

## Discussion

The present study aimed to explore clinicians’ perspectives of the incorporation of robotic dogs into therapy sessions for CSA survivors. This was achieved by investigating the clinicians’ use of the robotic dog in sessions, perspectives on the robotic dogs themselves, and beliefs about how the robotic dogs influenced treatment delivery. Overall, whilst generally positive, clinicians’ perceptions on the utility of robotic dogs in therapy sessions for CSA survivors varied depending on their own experiences in the study and the level of interaction their clients had with the robotic dog. A thematic analysis of interviews with clinicians revealed four main themes: (1) client characteristics that influenced their interest in the robotic dog; (2) clients’ behavioural interactions with the robotic dog; (3) robotic dog characteristics; and (4) clinicians’ use of the robotic dog.

### Key Findings

#### Client Characteristics That Influenced Their Interest in the Robotic Dog

Certain client characteristics (e.g., shyness, age, diagnoses/symptoms) seemed to influence whether and how clients showed interest with the robotic dog and, thus, influenced the perceived effectiveness of the robotic dog within sessions. Interestingly, two clinicians reported that clients who were shy were more likely to be drawn to the robotic dogs than those who were more outgoing. Shy clients were reported to have used the robotic dog as social support and were observed to be more comfortable talking to the robotic dog than the clinician and to seek comfort from the robotic dog. This is consistent with qualitative research on animal-assisted therapy (AAT) that found animals help facilitate a sense of comfort, confidence, and safety for children and young people in sessions (Flynn et al., 2020; London et al., 2020). Because CSA survivors may experience social withdrawal due to their trauma experience (Narang et al., 2019), robotic dogs may help them engage more successfully in the therapeutic process.

Contrary to previous research that has suggested socially assistive robots have been useful for children with ASD (Kim et al., 2013; Kozima et al., 2007), the clinicians in the current study reported that children with ASD were generally not interested in the robotic dog. In the present study, these clients reportedly knew that the robotic dog was not a real dog, therefore they did not want to interact with it. The differences between previous findings and the current study’s findings may be influenced by the age of participants and how directed they were to engage with the robots. Indeed, Kozima et al. (2007) research on children with ASD was based on clients who were aged between two to four years, which is much younger than those in the current study (i.e., 5 to 12 years). Moreover, Kim et al. (2013) used very directive prompts to encourage their participants to interact with their robot, whereas the clients in the present study generally led their interactions with the robotic dog themselves.

The clinicians in the present study generally reported that the older clients were, the less likely they were to be interested in the robotic dog. This is inconsistent with Björling et al. (2020) who found young people up to 18 years of age to be engaged with a socially assistive robot and to enjoy their experiences with it. Notably Björling and colleagues’ robot was more like a traditional metal robot, as opposed to the relatively more life-like (but still toy-like) robotic dog in the current study, with the former perhaps being interpreted as being more age appropriate by older children. Nonetheless, our results in this context are consistent with Broadbent et al. (2018) findings that children’s interest in socially assistive robots is influenced by their age.

#### Clients’ Behavioural Interactions with the Robotic Dog

The reported child/robotic dog interactions were varied, with many children being described as affectionate, caring, or nurturing toward the robotic dog. This is consistent with reported themes of nurturing, caretaking and attachment towards animals reported by research using AAT (Firmin et al., 2016; Flynn et al., 2020; Geist, 2013), suggesting that CSA survivors interact with the robotic dog in similar ways to young people in sessions with live animals. It also aligns with play therapy as a promising avenue to treat CSA (Narang et al., 2019).

In the present study, clinicians reported that clients would talk to the robotic dog when asked challenging questions either in response to or to avoid talking about certain topics with the clinician. Allen et al. (2022) also reported that when completing exposure tasks (e.g., talking about traumatic events), their participants (children and adolescents with trauma experience) would focus on the live dog in sessions. When this occurred, their clinicians reported it to be challenging to re-engage their participants. Unlike Allen et al.’s findings, the clinicians in the present study reported they were able to redirect clients’ attention back to the therapeutic task if needed or use the client’s avoidance as a signal that they were not ready to discuss the difficult topic (e.g., their experience of CSA). In a similar context, clinicians in our study reported finding the robotic dog to be less distracting than live therapy dogs, highlighting a strength of the robotic dog.

Whilst there appears to be no such mention of aggression involving socially responsive robots in prior research involving therapeutic settings, clinicians in this study reported the robotic dog was able to be used by clients in a more aggressive way (e.g., hitting or throwing it), allowing clients the opportunity to reenact their experiences of physical discipline, communicate their emotions through play with the dog, and gain the sensory benefits of physically expressing their emotions. Clinicians could use these interactions to gain an understanding of something the child had experienced or an emotion they were feeling. Clinicians could then turn those interactions into a learning experience for the client (e.g., psychoeducation on feelings). This highlights the benefit of utilising the robotic dog in this context, as sometimes, children who have shown aggression towards animals are excluded from DAT due to the potential risk on the animal’s welfare (Allen et al., 2022; Hartwig, 2017) and these children are, therefore, unable to gain the benefits from this type of therapy. Because externalising behaviours such as aggression are common amongst CSA survivors (Kendall-Tackett et al., 1993; Lewis et al., 2016), the robotic dog may be a way for these children to still experience a form of (robotic) DAT. The responsiveness of the robotic dog (e.g., reacting to noise) meant clinicians were better able to explain that the robotic dog may have feelings, compared to a toy, when providing, for example, psychoeducation. In this context, the robotic dogs may be more useful for delivering this type of psychoeducation compared to live dogs as welfare considerations are not a concern.

#### Robotic Dog Characteristics

Current findings indicate that there are some challenges when using the robotic dog in sessions, most of which stemmed from its characteristics. Most clinicians reported that clients could find the robotic dog's barking at times distracting and that such barking disrupted the sessions. This was overcome in sessions by the robotic dog being muted or turned off. Additionally, the limited nature of the robotic dog’s movements and responsiveness was a substantial weakness in its ability to be applied or used in more therapeutic situations, though its responsiveness was reported to be a strength when compared to non-robotic toys. Moreover, despite being advertised by *Joy for All* as soft and clinicians in the present study describing its fur as soft, the robotic dog was reported by one clinician as having a hard, metal body which negatively impacted its ability to be cuddled. At present, these challenges limit the range of the robotic dog’s utility in sessions, which could deter some clinicians from using it. Nonetheless, most of these limitations could conceivably be overcome with technological advancements. Indeed, there are a number of more advanced robotic animals on the market such as Tombot, the emotional support dog (see: https://tombot.com/) or Paro, the therapeutic robot seal (see: http://www.parorobots.com/), each of which comes with its own advantages (e.g., increased functionality such as responsive to voice commands) and disadvantages (e.g., high financial cost; limited research for use with children). It would be beneficial for future research to investigate which characteristics of a robotic dog would be important for use in therapy sessions for CSA survivors to facilitate the development of a purpose-built robotic dog for this population.

#### Clinicians’ Use of the Robotic Dog

The effectiveness of the robotic dog appeared to be partially influenced by the way in which the clinicians directed the client to use it. Most interactions between the client and the robotic dog were child-led; that is, the clinicians waited for the client to show interest in the robotic dog before using it. This indicates a substantial difference to how live dogs are typically actively introduced and used in sessions in DAT (O’Haire et al., 2015). Clinicians reported that drawing attention to specific toys in the room (including the robotic dog) did not fit with their therapeutic approach, further emphasising that the clinicians saw the robotic dog as a toy. However, it was suggested by a clinician that if they were more directive, the clients would have likely engaged more with the robotic dog, and it would have had more of an influence on reaching goals. Future research should aim to determine if this is indeed the case, but it is possible, given the cognitive, behavioural, and psychological impacts of CSA on survivors (Narang et al., 2019), which may make it more difficult for the children to even begin to play with, or work with, the robotic dog.

The present study’s results suggest the robotic dog was able to be integrated into a variety of therapeutic approaches, with reports of it being particularly useful in play-based therapies, an approach with limited, but positive, evidence for CSA survivors (Narang et al., 2019). The robotic dog was an especially beneficial tool when used for psychoeducation around consent, rapport development, and facilitating interactions between the client and clinician, with aspects of these being reported by three clinicians. While clinicians would have been able to provide psychoeducation and develop rapport in other ways, the robotic dog was a useful tool to aid this. The use of the robotic dog in aiding rapport building appears to be akin to the manner in which live dogs are used to facilitate the development of therapeutic alliances between children and clinicians in DAT (Hill et al., 2020). Given that the therapeutic relationship between clinician and client is estimated to account for around 30% of positive change in therapy (Hubble et al., 1999) and the significant challenges that CSA survivors have with developing rapport in therapy (Signal et al., 2017), the robotic dog provides a novel way to facilitate the development of rapport within this population.

### Theoretical Considerations

Whilst prior AAT or social robot research has not discussed, this issue extensively, children’s interactions with the robotic dog may be able to be interpreted through similar theoretical mechanisms of human–dog interactions (Barber et al., 2021), specifically, the social support theory (i.e., dogs facilitate social interactions between people due to the interactive nature of dogs), the biophilia hypothesis (i.e., people have an inherent desire to connect with living things and/or objects with life-like features leading to feelings of safety and other benefits), and neurobiological theory (i.e., human–dog interactions activate the oxytocin system, therefore reducing stress and enhancing social connection). Very briefly, children’s interactions with the robotic dog as reported by the clinicians (i.e., showing interest and positive affective behaviours such as treating it as a comfort object) seem to be consistent with social support theory, in that these interactions may increase the therapeutic alliance and rapport development. Such interactions may conceivably be via the involvement of oxytocin, although clearly future research would need to determine whether such biological reactions do occur. Although none of the clinicians deemed the toy to be overly life-like, the limited life-like features and responsiveness of the robotic dog may have contributed to facilitation of biophilia-related beliefs towards the robotic dogs. Moreover, it is possible that for children with ASD, the robotic dog was not life-like enough for them to want to interact with it. Nonetheless, the preference of some children to interact with the robotic dog rather than other toys and the reported utility of the robotic dog as opposed to other toys by clinicians may be related to its life-like features. Keeping the biophilia hypothesis in mind, it will be imperative that future robotic dogs used in therapy are designed with enhanced realism in mind. Determining which, if any, of these theories hold currency for explaining the therapeutic effects of animals and/or robots should be a priority of future research.

### Methodological Considerations and Future Directions

Considering the integral role of clinicians in the application and delivery of evidence-based interventions, the adopted methodology allowed for an investigation regarding the use of and practicality of using robotic dogs by obtaining input from those directly involved in the treatment of CSA survivors. This is important as it allows for the possibility of robotic dogs’ utility to be assessed in real world situations and provides a more nuanced understanding of how they can be used in sessions. Another strength of this study is that the participating clinicians utilised diverse therapeutic approaches as their treatment as usual. As such, the current findings may be applicable to a variety of therapeutic approaches, although further exploration is warranted. Moreover, the present study’s methodological rigor, via the adoption of Braun and Clarke’s (2006) widely recognised thematic analysis procedure when collecting and analysing the data, was a strength. Furthermore, the transcripts were deemed to have high credibility as clinicians were involved in checking the accuracy and interpretation of these documents (Thomas & Magilvy, 2011). Finally, the data displayed dependability in that the identified codes, themes, and interpretations were those of multiple researchers (Thomas & Magilvy, 2011).

Several limitations exist, however. Many of the clinicians had limited experience using the robotic dog, meaning that their reporting of its use in sessions may not have been as detailed as it might have been had they had more experience with the robotic dog. Reassessing clinicians’ experiences with the robotic dogs after a longer period, particularly after completion of treatment, would be beneficial. Moreover, while the small sample size of clinicians allowed for investigation of a previously unexplored area in detail, it also limits the extent of experiences that could be compared, reducing the transferability of the results (Thomas & Magilvy, 2011). Similarly, only two clinicians reported having experience with *any* live animal assisted-therapy, which disallows most of the sample from being able to make meaningful comparisons and hence suggestions involving how robotic animals differ from their live counterparts. Ideally, future researchers will recruit a larger sample of clinicians with live animal assisted therapy experience to gain a wider and more diverse understanding of how they compare to robotic dogs with CSA survivors.

None of the participating children in our study identified as being from Aboriginal and/or Torres Strait Islander, or culturally and linguistically diverse backgrounds. Different cultures often have different views of dogs; for example, canines are strongly featured in Aboriginal cultures within Dreamtime stories, as well as for companionship, in a way that is fundamentally different from Western relationships with dogs (Constable et al., 2010; Glynn-McDonald, 2022). Given that individuals’ interactions with robotic dogs can be influenced by their beliefs about live dogs (Barber et al., 2021), it is possible that different cultural values and perceptions around dogs could influence CSA survivors’ interactions with robotic dogs. This highlights the need for future research to include child participants from more diverse cultural backgrounds.

Finally, whilst our study focused on CSA survivors, it is important to acknowledge that the clinicians showed heterogeneity in terms of (i) the children treated (e.g., whether children had diagnoses such as ASD, how engaged children were with clinicians, etc.), along with (ii) their therapeutic style (e.g., narrative therapy, child play centred therapy, etc.). Such factors may have influenced their responses regarding the thematic analyses performed, and as such, future researchers should plan to account for the analysis of such factors to see what effect – if any – they may have.

The present study achieved the aim of exploring this novel research area from the clinicians’ perspectives, yet information from the children who attended the therapy sessions with the robotic dog themselves was not considered. It would be beneficial for future research to study CSA survivors’ perspectives of the robotic dog in sessions to further improve our understanding of how the robotic dogs are perceived. Similarly, future researchers could conduct quantitative analyses of clients’ presentations to see if the incorporation and use of the robotic dog have a significant impact on therapeutic outcomes (e.g., trauma symptoms) as well as exploring any impacts on clients’ retention/engagement in sessions. Finally, it would be beneficial to directly compare the effectiveness between robotic and live dogs for treating CSA survivors to better understand whether, and how, robotic dogs have the potential to be a viable alternative or additional option to live therapy dogs for CSA survivors.

### Practical Implications and Conclusion

Whilst Burr et al. (2023) indicated that clinicians generally believed that robotic animals are a potential alternative to AAT, to date no such research addressed clinicians’ perspectives of this for use with CSA survivors specifically. As such, the obtained findings have practical implications for contexts involving CSA survivors, although we are not claiming such findings will only be specific to CSA survivors. Foremost, our preliminary evidence now demonstrates that there are potential benefits in using robotic dogs for CSA survivors, a population that warrants particular concern for improved treatments given they are less likely to benefit from treatment than children who have not experienced CSA (Cohen & Mannarino, 2015; Signal et al., 2017), and due to the lifelong psychological and psychosocial consequences of CSA (Nagtegaal & Boonmann, 2022). Those clinicians considering using robotic dogs as an adjunct in treating CSA survivors should do so knowing that they may potentially be particularly effective for developing a therapeutic alliance and can be especially useful for psychoeducation. Moreover, the robotic dog was generally well received by child clients. For example, the robotic dog was used as a comfort object by most children, suggesting that the presence of a robotic dog may possibly help CSA survivors feel safer in a therapeutic space, which is particularly important for these clients. These potential benefits cannot be overlooked given the high dropout rates of CSA survivors in therapy and numerous other challenges this population faces when engaging in therapy (Murphy et al., 2014). There appear to be very few potential disadvantages related to using robotic dogs with this population, the most serious of which were generally able to be overcome (e.g., by muting the robotic dog if it were barking). As the field of socially assistive robotics continues to progress, it is hoped that robotic animal therapies will usher in meaningful improvements in treating CSA survivors.

## Data Availability

Please contact the corresponding author to request access to the datafile.

## Footnotes

1 Unless otherwise noted, from here on, CSA survivors refers to sexual abuse survivors who are still children.

2 Notably, Clinician 1's clients (see Table 2 below) did not seemingly show interest in the robotic dog after its introduction. Therefore, questions pertaining to the specific use of the robotic dog in sessions were not asked of her, and hence her interview time of 14 min.
